# Recognition of Non-Manual Content in Continuous Japanese Sign Language

**DOI:** 10.3390/s20195621

**Published:** 2020-10-01

**Authors:** Heike Brock, Iva Farag, Kazuhiro Nakadai

**Affiliations:** 1 Honda Research Institute Japan Co., Ltd., Wako-shi, Saitama 351-0188, Japan; nakadai@jp.honda-ri.com; 2 Faculty of Sciences and Engineering, Saarland University, 66123 Saarbrücken, Germany; ivafarag@gmail.com

**Keywords:** sign language, learning systems, motion segmentation, signal processing, gesture information retrieval, neural networks

## Abstract

The quality of recognition systems for continuous utterances in signed languages could be largely advanced within the last years. However, research efforts often do not address specific linguistic features of signed languages, as e.g., non-manual expressions. In this work, we evaluate the potential of a single video camera-based recognition system with respect to the latter. For this, we introduce a two-stage pipeline based on two-dimensional body joint positions extracted from RGB camera data. The system first separates the data flow of a signed expression into meaningful word segments on the base of a frame-wise binary Random Forest. Next, every segment is transformed into image-like shape and classified with a Convolutional Neural Network. The proposed system is then evaluated on a data set of continuous sentence expressions in Japanese Sign Language with a variation of non-manual expressions. Exploring multiple variations of data representations and network parameters, we are able to distinguish word segments of specific non-manual intonations with 86% accuracy from the underlying body joint movement data. Full sentence predictions achieve a total Word Error Rate of 15.75%. This marks an improvement of 13.22% as compared to ground truth predictions obtained from labeling insensitive towards non-manual content. Consequently, our analysis constitutes an important contribution for a better understanding of mixed manual and non-manual content in signed communication.

## 1. Introduction

Systems that process and understand expressions in Sign Language (SL) have a great potential to facilitate daily life of individuals that are deaf or hard of hearing. Nevertheless, to date no universal system could be found that would be accurate, reliable and applicable to general, daily use. This is due to a number of complexities specific to SLs.

First, SLs are visual languages and impose specific sensing requirements to obtain meaningful representations of the moving joint trajectories through time and space. As such, machine learning data cannot be obtained as easily as in other domains. In order to provide ubiquitous application systems, it is reasonable to focus on simple sensing devices, may it be video cameras [1], the Microsoft Kinect [2,3] or the Leap Motion [4]. High recognition rates can be achieved for isolated signs [5,6,7]. However, it is very challenging to recognize the content of continuous sentence expressions in more realistic settings. Here, lexical items might merge into each other without clear visual separation, appear in unbalanced frequency, or undergo morphological changes based on speed, content and personal style of a signer. Lastly, signed expressions incorporate a wide range of specific linguistic features. Examples are Non-Manual Expressions (NMEs) and spatial and contextual Classifier Predicates (CP). These features may vary with the context of a conversation, and can hardly be put into generic concepts [8] for subsequent retrieval from the collected sentence data. As a result, it is necessary to develop specialized continuous Sign Language Recognition (CSLR) approaches that are equally robust towards morphological variations, and sensitive towards the special characteristics of a SL.

In this work, we particularly aim to investigate the latter aspect, in order to foster the development of communication assist systems that understand detailed non-manual information. For this, we introduce a novel recognition system based on skeletal information of body joints obtained from a single camera video data. This makes our system lightweight and faster to train as compared to a system solely based on video data. In particular, we make use of the publicly available OpenPose system [9,10,11,12] to infer upper body, finger and facial joint positions of signed video content. These two-dimensional data points are then post-processed and utilized as input in the proposed CSLR interface. Here, the idea is to ease the burden on the network training by a two-staged interface. Rather than simultaneously learning the temporal segmentation and gesture content, we first separate relevant content within the continuous expressions. The subsequent recognition step should then be more sensitive to small subtle differences within the data, which often characterize content-relevant NMEs.

To justify the applicability of our proposed system, we evaluate it with our own collection of sentence expressions in Japanese SL. Under a strict evaluation that takes misclassification of non-manual content into account, we achieve an average Word Error Rate (WER) of 15.71%. In particular, we are able to distinguish various types and intensities of NMEs and CPs. Therewith, the main contributions of this work are as follows:

- Introduction of a sign segmentation method for the acquisition of robust sentence split proposals and its subsequent post-processing, followed by a profound discussion and evaluation.
- Evaluation of multiple skeletal feature-based Convolutional Neural Network (CNN) architectures for classification of word segments with and without NMEs.
- Extensive evaluation of the obtained CSLR system outputs including an analysis of the impact of NMEs on the overall recognition accuracy.

## 2. Background

A number of learning systems utilizing both staged and combined strategies to address the problem of CSLR from video data have been reported. Exploring the problem under conventional machine learning, 2-stage systems that first segment words and subsequently classify the obtained segments were popular in the early stages of research [13,14]. Combined systems mainly evolved with the technological possibility of end-to-end learning. They aim to unify the two problems into one model architecture in order to prevent error accumulation caused by imperfect temporal segmentation. State-of-the-art techniques with the best achieved WER all utilize the RWTH-PHOENIX-Weather corpus featuring 7k sentence expressions of weather forecasts in German Sign Language [15].

Deep neural network architectures could considerably improve the quality of CSLR systems within the last years. The first deep network proposed by Koller et al. [16,17] utilized a CNN as feature extractor for classification of hand shapes on top of a segmentation step based on a Hidden Markov Model (HMM) and Recurrent Neural Network with Long-Short-Term Memory (LSTM) for sequence modeling. The network reached a WER of 30.0% in a single signer, and 38.8% in a multi-signer scenario with the PHOENIX data set. Moreover, it achieved a WER of 7.4% under a more controlled data set referred to as SIGNUM [18]. Using hand only information, a sequence-to-sequence learning approach based on Connectionist Temporal Classification (CTC) and SubUNets by Camgoz et al. [19] achieved a WER of 48.2%. Cui et al. [20] developed a three-staged learning model with CTC-based sequence alignment, for which they report a WER of 38.7% in the multi-signer scenario. A hybrid model for temporal sequence alignment without segmentation was proposed by Huang et al. [21]. This model is based on a 3D CNN that is combined with a Hierarchical Attention Network, an extension of recurrent neural network cells. Here, WER for an independent data set of Chinese SL was 17.3%, whereas the best WER for the PHOENIX data set was 38.3%. Camgoz et al. [22] explored the problem of sign language translation as inspired by neural machine translation frameworks for spoken languages. Their proposed architecture combines spatial video and word embeddings in an attention-based encoder-decoder network. By this, the authors were able to learn gloss output from video input without using an intermediate hand-shape classifier framework. The latest network of Cui et al. [23] employs an end-to-end iterative learning process utilizing a temporal alignment proposal without segmentation. Best WERs are reported as 24.43%. The most recent work of Koller et al. [24] extended the hybrid CNN-LSTM-HMM architecture to include mouth shape and hand shape. As a result, recognition accuracy could be improved to a WER of 26.0%. Lastly, Papastratis et al. [25] showed that that the accuracy of sentence predictions could be further enhanced by a cross-modal learning approach that leverages text information.

### Nmes and Japanese Sign Language

Learning gloss-like representations from sequences of image data in an end-to-end fashion requires the availability of sufficient data. However, as Koller et al. [17] point out, word frequency in the PHOENIX data set is highly imbalanced, and numerous singletons (i.e., words that appear only once) impede the learning of a strong classifier. This might particularly affect the distinction of non-verbal expressions and linguistic information that are not frequently used, but convey specific detailed context information. Moreover, corpus annotation does not focus on the recognition of NMEs and CPs within continuous sentence expressions. This impedes the analysis of learning systems customized to respective linguistic aspects of a SL.

A more linguistically focused data set is the American Sign Language data set used by Ye et al. [26]. However, as we aim to ultimately develop a communication assist system for Japanese deaf or hard of hearing users, we decide to perform the following analysis on our own constructed set of sentence expressions in Japanese Sign Language (JSL) [27]. JSL is a distinct language of its own culturally engraved morphology, grammar and expressivity. Taiwanese SL—which is thought to have evolved from JSL—is its linguistically closest language [28]. As such, JSL differs considerably to German or American SL. One illustrative example is the sign ‘eating’: in JSL, it is depicted by two chopsticks that move rice from a bowl to the mouth, whereas in German and American SL it resembles the process of eating a sandwich. Similarly, NMEs in JSL follow a different cultural understanding [29]. This makes them very subtle and less expressive than other SLs. In 2000, Sagawa and Takeuchi [30] developed a system for recognition of short sentences of 3 to 6 signs, which was able to understand pointing gestures within their contextual context from head movement. Since then, no similar works have been reported that utilize JSL sentences with NMEs. Therefore, we hope our work to fill a gap in current CSLR for JSL communication assist systems, while simultaneously also offering the potential to provide new insights into technological development for different SLs.

## 3. Cslr System Pipeline

Previous efforts to implement a bidirectional sequence-to-sequence system from joint position data [31] show that it is very difficult to learn a working system for the given data set due to its high variability induced by the presence of linguistic features. For this reason, we perform our following analysis under a two-stage system with temporal segmentation. As a consequence, word boundaries within a continuous sign movement flow are detected independently of the specific word content.

**Figure 1:**
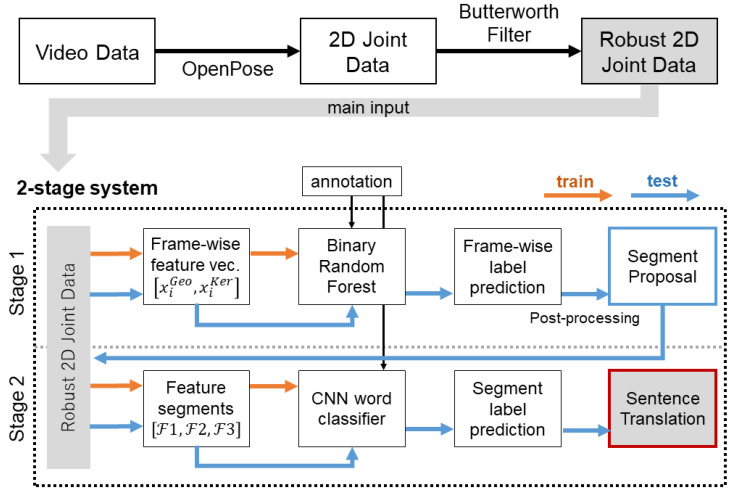
Overview of the complete system outline with the proposed two-stage recognition pipeline.

Following the system flow shown in Figure 1, the general pipeline of our proposed CLSR is as follows: as an initial step, we extract the two-dimensional data points of upper body, finger and facial joints from our sign video frames. After data smoothing and interpolation of missing joint positions we then proceed to the two principal recognition stages. In the first stage, we determine word boundaries within the signed expressions utilizing the output of a trained binary frame-wise Random Forest (RF) classifier. Sentence splits are proposed on the base of spatial and non-linear relations between consecutive frames and the confidence values of the RF. These data segmentation proposals next undergo a post-processing step for refinement and enhanced robustness. In the second stage, the post-processed segmentation proposals are used to extract single data segments from the extracted 2D body joint positions accordingly. A specialized CNN architecture is then utilized to classify each segment cut on the base of the skeletal data. Lastly, resulting word labels are re-concatenated in their order of occurrence and a final sentence prediction in gloss annotation is obtained. Making use of tracked joint locations, our work most closely resembles the architecture discussed by Joze et al. [32] that uses LSTMs to learn characteristics of signed words. However, whereas the authors focus on the effect of training data on the recognition accuracy, we focus on the learning of non-manual content information in the following.

### 3.1. Stage 1: Sentence Segmentation

We train a binary classifier for frame-wise evaluation of single data frames as proposed by Farag and Brock [33]. The original method evaluates 3D motion capture data frames of the upper body and finger joints with a RF based on its spatio-temporal surrounding. For the current work, we adapt the method to the reduced dimension of the underlying sign video. Facial movement is considered as insignificant for the identification of transitional phases, and hence omitted in this stage. Alternative classifiers and neural networks could be employed instead of a RF, but did not lead to performance improvements in preliminary explorations. This is most likely due to the fact that we only observe immediate short-term dependencies of few neighboring frames. Such a time span might be insufficient to extract relevant information for modern classifiers like CNNs. For this reason, we keep the original RF approach.

The RF labels every frame as either transitional movement (class 0) or sign movement (class 1). As a result, without any assumptions, we obtain an initial accurate temporal segmentation of pure activity sub-sequences. This segmentation is sensitive to the spatial as well as the velocity components of the motion. Moreover as described in the following, the robustness of the resulting split proposals can be enhanced utilizing post-processing on the base of the classifier’s confidence values.

#### 3.1.1. Frame-Wise Classification

For each frame *i* in the motion sequence, we compute a feature vector $x_{i}$ which depicts phases of temporal and directional transformation. $x_{i}$ consists of a general geometric feature descriptor $x_{i}^{\mathrm{Geo}}$ that represents the spatial (and angular) relations between body joints over a certain time span, and an additional kernel descriptor $x_{i}^{\mathrm{Ker}}$ that represents their non-linear relationship obtained from a Laplacian kernel transformation of $x_{i}^{\mathrm{Geo}}$.

From any representation of angular joint relations, $x_{i}^{\mathrm{Geo}}$ can be computed as spatio-temporal representation $x_{i}^{\mathrm{Geo}}=\left[f_{i-w_{g}},\cdots f_{i},\cdots f_{i+w_{g}}\right]$ around a window $w_{g}$ of neighboring frames. Joint relations shown to provide reasonable segmentation results are the combination of angular and distance features between line segments of pairs of joints introduced in [33], or the joint angular displacement transformation by Kumar et al. [34]. Another possible variation could be to utilize the point of intersection between selected line segments as reference point for a subsequent computation of joint or inter-segment angular displacements. To find the best $x_{i}^{\mathrm{Geo}}$, we evaluate the performance of the RF with respect to all three variations adapted to the given setting: (a) the 2D version of the original line segment relation features (in the following referred to as LS), (b) its modified version encoding joint angular distances with respect to the point of intersection between the line segments (in the following referred to as LS-IS), and (c) a joint angular displacement transformation (in the following referred to as JAD) utilizing the same joint pair combinations as LS and LS-IS.

For all three variations, the frame-wise kernel matrix for a motion sequence of *t* frames and its corresponding geometric feature matrix F is defined as

(1) $$\begin{array}{c} K=\phi {\left(F\right)}^{⊤}\phi \left(F\right)\in R^{\;t\times t}, \end{array}$$

where $k_{i,j}$ characterizes the similarity between the spatial feature vectors $f_{i}$ and $f_{j}$ of frames *i* and *j* in terms of the kernel function $\phi {\left(f_{i}\right)}^{⊤}\phi \left(f_{j}\right)$. The kernel feature vector for a given frame *i* and window size $w_{k}$ is then defined as the flattened upper triangular sub-kernel $x_{i}^{\mathrm{Ker}}=\mathrm{triang}\left[\ldots K_{uv}\ldots \right],\;\forall \;u,v\in \left[i-w_{k},i+w_{k}\right]$. As discussed in [33], the idea here is to derive further high-level understanding of skeleton movement dependencies over time.

Following the original work, we apply the proposed window sizes $w_{g}=2$ around frame *i* to concatenate all corresponding feature vectors to $x_{i}^{\mathrm{Geo}}$, and $w_{k}=10$ around frame *i* to build $x_{i}^{\mathrm{Ker}}$. The concatenated frame-wise data representation $x_{i}=\left[x_{i}^{\mathrm{Geo}},\;x_{i}^{\mathrm{Ker}}\right]$ is computed for each frame within a given training and test set. The resulting classification label for every feature vector is then used to determine an initial sentence segmentation proposal as the sequence vector $sp_{ini}$. Consecutive frames classified as belonging to class 1 are interpreted as the segments of interest, also holding information about their start and end points. The frames classified as class 0 and positioned in-between different segments are considered non-gestures or transitional frames.

#### 3.1.2. Confidence-Based Split

The RF classifies each frame *i* on the base of its confidence $c_{i}$ about the respective frame’s affiliation to class 1. This means that every frame with confidence $c_{i}\geq 0.5$ is labeled as 1, and 0 otherwise. Ideally, every motion frame would be assigned to class 1 and every transition frame would be assigned to class 0. However in practice, it is nearly impossible to obtain such perfect split within a natural signing flow. To obtain a robust segmentation of sign and transitional movements, we refine the actual sentence segment prediction $sp_{ini}$ with the following signal-based post-processing.

The distribution of $c_{i}$ values for a frame belonging to class 1 over all motion sequence frames t defines a temporal prediction curve *c* with $c_{i}\in c\;\forall \;i\in \left[1,2,\cdots ,t-1,t\right]$. Over the progression of a signed expression, *c* follows a fundamental sine-like pattern: parts of high and low confidence take turns on the base of the reciprocal occurrence of transitional and sign movements. We therefore compute a smooth version $c_{A}$ of *c* with a Gaussian filter of kernel deviation $\sigma_{A}=5$. Here, the idea is that $c_{A}$ is robust to smaller erroneous parts of mislabelling within multi-directional words or complex movements. As such, $c_{A}$ eliminates disturbances caused by classifier predictions inconsistent in their initial confidence rating. The values of $c_{A}$ can then be used to obtain a smoothed segmentation proposal $sp_{smg}$.

Sources of noise (e.g., stutter, fast movement between two separate words or the signing of complex, multi-directional words) might both add or remove peaks to the basic evolution of *c* that cannot be represented in $sp_{ini}$ or $sp_{smg}$. Since the main objective of our work is to investigate the recognition of NMEs, we aim to obtain the most robust sentence splits as possible. Therefore, we include additional information on the number of sign words occurring per sentence pattern. Here, it should be noted that this strategy is an optional, minor fine-tuning process which can be omitted in the absence of respective word count information. As such, it also does not significantly influence the following system evaluation.

**Figure 2:**
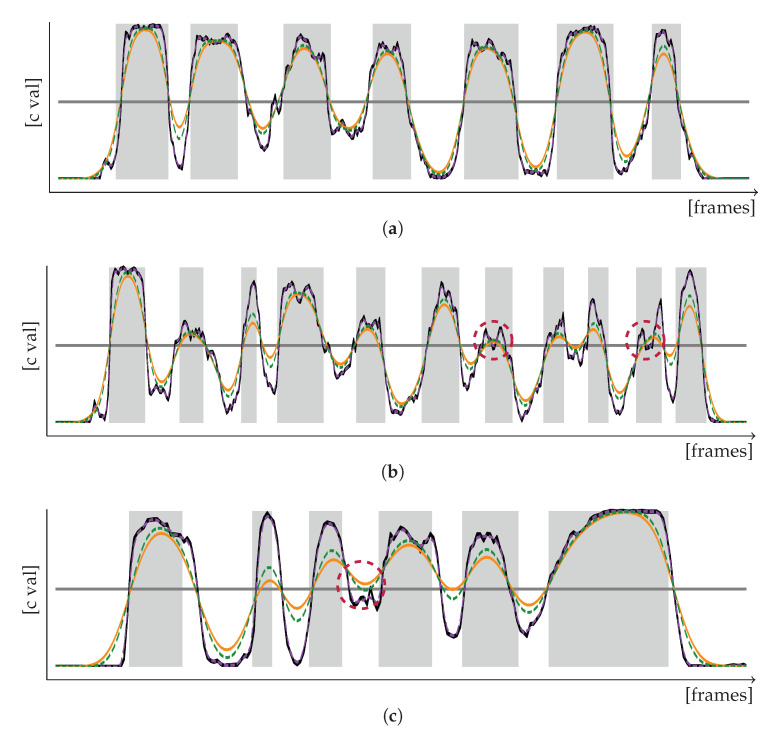
Confidence curves *c* (black, solid) and their respective smoothed versions $c_{A}$ (orange, solid), $c_{B}$ (green, striped) and $c_{C}$ (purple, striped) of sentence-based label predictions in relation to the binary decision threshold of 0.5 (gray, solid) and the actual segment boundaries of the signed expression. (**a**) The confidence curves follow a sine-like pattern closely related to the ground truth segmentation. (**b**) $c_{A}$ and $c_{B}$ are more robust to inter-word misclassifications caused by minimal peaks of *c* falling below the threshold (red circles, dashed). (**c**) Strong smoothing of $c_{A}$ can smooth out peaks and result in information loss (red circle, dashed).

For the optional segmentation refinement, we generate two additional, modified versions from the prediction curve *c*: a mildly smoothed version $c_{B}$ of *c* by applying a Gaussian filter of kernel deviation $\sigma_{B}=4$, and a weakly smoothed version $c_{C}$ of *c* by utilizing a Savitzky-Golay filter of window length $w_{C}=3$ and polynomial order $n_{C}=3$. Plotting the filtered confidence curves (Figure 2), one can see that strong Gaussian smoothing results in confidence curves which are more robust to smaller erroneous parts (Figure 2b), but which also miss significant information in parts of quick signing and flowing transitions between signs (Figure 2c). To account for such information loss, we determine the number of word segments given by $c_{A}$ and compare them to their actual word count. All expressions whose word count is smaller than the sentence content then undergo a more detailed signal-based segmentation check. This enhances the probability that noise-induced $c_{i}$ peaks which were correctly smoothed out in $c_{A}$ will be left unconsidered.

To start, we identify all significant peaks of *c* that were smoothed out in $c_{A}$, but would remain present in $c_{C}$. In concrete, we detect all maximal peak locations $p_{max}$ and all minimal peak locations $p_{min}$ of $c_{A}$ and identify their labels as given by the respective $c_{Ai}$. Next, we identify the correlating reference locations *r* of maximal (for $p_{max}$) and minimal (for $p_{min}$) $c_{Ci}$ values within a window $w_{p}$. Here, $w_{p}$ is defined as the area between the greatest lower bound (glb) and the least upper bound (lub) of $c_{A}$ and $c_{C}$ around every peak in $p_{max}$ and $p_{min}$. For maximal peaks, this for example holds

(2) $$\begin{array}{cc} r & =\underset{i\in w_{p}}{\mathrm{argmax}}c_{Ci} \end{array}$$

with $w_{p}=\left[\mathrm{lub},\cdots ,\mathrm{glb}\right]$ for glb $=c_{Ai}\wedge c_{Ci}$∀ i ∈[1,⋯,p] with $p\in p_{max}$, and lub $=c_{Ai}\vee c_{Ci}$∀ i ∈[p,⋯,t] with $p\in p_{max}$. Lastly, we replace all $c_{A}$ labels within the specific $w_{p}$ with the labels of $c_{Cr}$. This gives us a refined segmentation proposal $sp_{med}$.

Based on the number of mismatches *m* between the word count of $sp_{med}$ and the actual known sentence word count, we further refine the proposal in case of m≠0. Next, we target to retrieve all parts of *c* that are most likely to be misclassified by the RF, namely the locations of minimal peaks $p_{min*}$ with $c_{i}\geq 0.5$ and the locations of maximal peaks $p_{max*}$ with $c_{i}\leq 0.5$. Similarly to the previous procedure, we utilize windows of intersection between two smoothed confidence curves around all peak values of interest. To retrieve peak values that were smoothed out by the high filter value $\sigma_{A}$, we determine the points of intersection between $c_{C}$ and $c_{B}$. We find the *m* most likely mislabeled locations following the definition given in Equation (2). Lastly, we replace their determined respective window areas with either class 0 or class 1 labels accordingly. This provides us with the final segmentation proposal $sp_{fin}$.

### 3.2. Stage 2: Word Classifier

We train a CNN to classify single word segments extracted from the video-based joint position data. The CNN is adapted specifically to the automatic word segmentation technique, i.e., we need to annotate the proposed word segments to their corresponding word class. We use a simple intersection analysis between word boundaries based on the provided ground truth annotation. If there is an overlap, we adopt the word label and otherwise we annotate as unknown. Once the temporal word boundaries of a test sentence are determined, we then cut the sequence into its according word segments and classify their respective word labels.

#### Skeletal Cnn

Skeletal data does not contain any intrinsic image-like structures. For this reason, we aim to transform the 2D body positions into a compact and meaningful data representation that emphasizes boundaries between time and space in a pixel-wise manner. Following a similar strategy as recent successful works from the human activity recognition field [34,35,36], we test three different representations of inter-joint relational changes under two different feature combinations that we designate as feature set $F1_{BH}$ respectively $F1_{BHF}$, $F2_{BH}$ respectively $F2_{BHF}$, and $F3_{BH}$ respectively $F3_{BHF}$. Skeletal data obtained from the post-processing step described in Section 4.2 builds our basic input data for the following three data transformations, whereas the feature indexation stands for: B body, H hand and F face. This means that for every feature representation we build one version that only constitutes of body and finger data, and one version that constitutes of body, finger and facial data.

To compute the feature representations, we first set all available joint positions into relation to a reference joint. For all pose joints, we define this reference to be the chest joint inferred from the known shoulder and hip positions. Finger joints are set into relation to the corresponding wrist joint, meaning the right wrist for all right finger joints and the left wrist for all left finger joints. The neck build the reference point for all facial joints. By simple data scaling and normalization, we then obtain our first feature set F1. Next, we set all joint positions into relation to their previous joint within the kinematic chain, whereas the inferred chest joint serves as origin of all kinematic chains respectively root joint. We again scale and normalize the data and obtain our second feature set F2. Based on their success in sentence segmentation, we compute a third feature set F3 from JAD features. We consider all available joint data except the eyes, ankles and a second chest joint.

## 4. System Implementation and Training

### 4.1. Data Set

We evaluate our proposed approach on an extended collection of the DJSLC corpus introduced in [27]. The extended corpus comprises of 1432 sequential sign expressions (of which 930 are sentence expressions with a length of 6 to 13 signs, and 502 are short phrases with a length of 3 to 6 signs) in JSL conveyed by a single, native signer. During data recording, the movement of the signer was made available utilizing a high-resolution optical motion capture system and a front-view 4 K video camera capturing the upper body movement of the signer. Since we focus on single video-camera data, we only make use of the set of video data in this work. Further details on the data collection process can be found in detail in [27].

**Figure 3:**
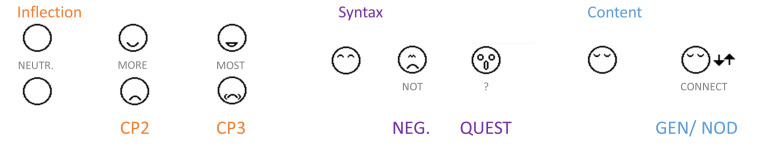
Symbolic description of the NMEs of JSL investigated in this work. Left: NMEs for CP2 and CP3 indicating adjective inflection. Middle: syntactic NMEs to express negations or questions. Right: NMEs carrying contextual information.

The sentence structure of the corpus was specifically designed as a way to allow for both the learning of actual life-like content and the analysis of advanced system development for linguistic analysis. It therefore contains a high number of NMEs with subtle variations in signing to convey adjective inflection, syntax or contextual references. In concrete, we aim to recognize the following specific linguistic features as they are also illustrated in Figure 3:

- CP2: non-verbal expression (commonly facial expression) to convey inflection of an adjective (level 2, comparative).
- CP3: non-verbal expression (commonly facial expression) to convey inflection of an adjective (level 3, superlative).
- Neg.: non-verbal expression (commonly slight movement of the head and upper body accompanied by a sad facial expression) used to express rejection or negation.
- Quest. (?): non-verbal expression (commonly slight movement of the head and upper body accompanied by a surprised facial expression) used at the end of a sentence to form a question.
- Nod: specific types of head movement used to connect two sub-sentences, which are either aligned or in logical contrast to each other.
- Gen.: specific head movement used as contextual information to express genitive references, such as Mr. Yamamoto’s wife.

**Figure 4:**
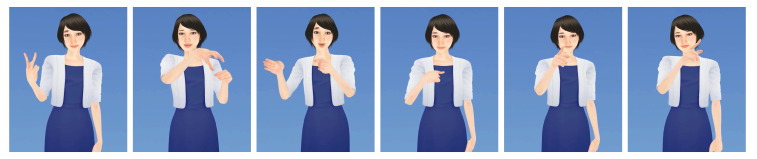
Sign avatar visualization of some of the JSL items for spatial and contextual references present in the corpus. From left to right: CP for two people (‘CP (2ppl)’), CP for places (‘CP (P)’), CP for buildings (‘CP (BL)’), pt1, pt2 and pt3.

Moreover, the corpus contains a number of spatial and contextual references that can influence the morphology of preceding or succeeding lexical items (sign classes), and hence further complicate the recognition task. In the given data collection the following references, of which some are also illustrated in Figure 4, are present:

- CP (item): Classifier Predicates used to convey a conceptual information ’item’, such as locations or shapes of things.
- pt1: reference to first person (oneself).
- pt2: reference to second person (conversation partner).
- pt3: reference to third person, object or location.

The sentences and phrases within the DJSLC contain 203 different lexical items, of which a high number can change their detailed, contextual meaning when combined with any of the previous NMEs. One example are comparative and superlative variations of an adjective, such as ‘tasty’, ‘very tasty’ (‘tasty (CP2)’) and ‘extremely tasty’ (‘tasty (CP3)’). Another example are CP items that describe semantic concepts, and build complex meanings in combination with surrounding words (e.g., the CP for buildings expresses the meaning ‘department store’ once combined with the sign for ‘buy’). Consequently, it might be meaningful to treat these sign and NME combinations as distinct motion classes. For this reason, we define two different sets of word classes that define the NME-sensitivity of the evaluated recognition system in the following: one basic set L1 consisting of all 203 basic sign classes, and an extended set L2 consisting of 273 sign classes, which includes the basic classes and all additional occurring NME combinations.

All training and system evaluation described in the following is performed under an 8-fold cross validation scheme, in order to make best use of all available signed expressions and their intrinsic variations in signing. This strategy furthermore helps to keep the evaluation free of any randomization bias.

### 4.2. Fundamental Data Preparation

Basic data preparation (Figure 1, top row) serves as the foundation for the staged recognition pipeline. We first obtain 2D joint positions by running the OpenPose framework. Our choice of OpenPose is influenced by the high number of body joints available for tracking, including face and finger joints. However, it should be noted that it is also possible to utilize alternative tracking frameworks. Missing data samples of every feature stream are then filled using a simple 1D linear interpolation. Lastly, all extracted joint trajectories are smoothed with a Butterworth filter. McDonald et al. [37] showed that it is reasonable to adapt smoothing filter values to the varying joint movements of SL motion capture data. We therefore assign joints of similar motion speed into separate groups of specific filter settings. These are strong filter values (cutoff frequency 1 Hz with a filter order of n=1) for the nearly static hips, knees, and the top of the head; strong medium filter values (cutoff frequency 1 Hz with a filter order of n=2) for the slightly moving shoulder and head joints; weak medium filter values (cutoff frequency 6 Hz with a filter order of n=2) for the elbow, all finger, and all facial joints; and finally weak filter values (cutoff frequency 8 Hz with a filter order of n=3) for the fast moving wrists (Table 1).

### 4.3. Stage 1 Segmentation Classifier

To identify the best feature set for the segmentation task of the first stage, we train three independent RF models for the LS, LS-IS and JAD feature representations under the chosen 8-fold cross validation scheme. Each tree is trained on a sub-sample from the original data set generated by bootstrapping with 300 decision trees of balanced class weights.

### 4.4. Stage 2 Word Classifier

We test multiple variations of a CNN under two distinct word class specifies. The first classifier type only evaluates hand shape and movement based on L1, and the second also includes subtle information as defined by L2. Combining the respective classified labels into one sequence, we can then compare system performance under the presence of NMEs in the following. For both L1 and L2, the chosen CNN variations are an architecture with two pairs of one convolutional and one pooling layer each followed by two fully connected layers, an architecture with three pairs of one convolutional and one pooling layer each followed by two fully connected layers, and their respective deeper versions with a second convolutional layer before each pooling layer. The size of the convolutional kernel was set to (5 × 5), and the pooling kernel was set to perform max pooling with kernel size (2 × 2). The number of kernel features per layer were (16) and (32) for the convolutional layers and (1024) for the fully connected layers. All network hyper-parameters were chosen in consideration of standard practices, and hence constitute some of the most basic CNN architectures as frequently employed in closely-related research domains [6,38,39,40]. For all feature set and word class set combinations, the most shallow architecture offered the best performance on average over all cross-validation folds, whereas its accuracy was ranging around ±90% in all cases. With the main goal of implementing a full working CLSR system, we consider the achieved accuracy as sufficient and do not further extend hyper-parameter tuning. Consequently, we continue our investigation with the respective best-performing four-layered network structure, and do not discuss the remaining architectures in detail.

Both feature sets F1 and F2 are brought into one-dimensional image-like structures. For feature combination BH, this image is of size 120 × 156 with 120 being the number of features and 156 being the maximal length of a sign. Similarly, the image size for feature combination BHF is 260 × 156. For those few sign annotations that are of higher length than 156 frames, we cut off any over length frames. Sign annotations shorter than 156 frames are padded along the right margin. For feature set F3 we follow the proposed strategy of Kumar et al. [34] and further transform all segments into three-dimensional joint angular displacement maps (JADMs) of standardized size using bicubic interpolation and a simple colormap transform. Considering the size of the data input before rescaling, we interpolate every word segment to be of size 256 × 128 with 256 being the modified feature size and 128 being the modified word length.

## 5. Analysis

### 5.1. Stage 1 Word Segmentation

We use the trained RF model to classify the frames of unseen video sequences and evaluate the performance on average over all 8 folds of the cross-validation. We report the performance of all models with respect to accuracy, precision, recall and F1-score in terms of correct binary detection of all class 1 frames in Table 2.

We can see that the modified LS-IS version of the LS features achieves better results than the initially proposed LS features, but that the JAD features achieve better results than the LS-IS features. While the LS features appear to be good descriptors for noise-free 3D skeleton information [33], the JAD features show a superior performance for the realistic setting of 2D video data. A chi-square based McNemar test between the frame-wise predictions confirms our observations. Here, the predictions of RF${}_{\mathrm{LS}-\mathrm{IS}}$ differ from the predictions of RF${}_{\mathrm{LS}}$ with strong statistical significance (*p* = 155.22), while they also differ from the predictions of RF${}_{\mathrm{JAD}}$ with very strong statistical significance (*p* = 13844.80). One reason for the better performance of the JAD-based features might be their higher robustness against noisy data points: a small perturbation of a joint position in the coordinate system could have a more significant effect on the derived line segments under LS and LS-IS. In the following, we therefore choose to use the JAD-based movement descriptors as well-suited data transformation for our practical setting of imperfect and noisy data conditions.

We refine the initial RF${}_{\mathrm{JAD}}$ in a random grid hyper tuning process and include the Brier score as additional quality metric for the selection of the final model parameters. Here, the idea is that the better a RF model is able to differentiate between both classes, the more reliable and less prone to errors $sp_{fin}$ should be. The ideal Brier score would be 0.00. As a result of the hyper parameter tuning, we obtain a final classifier RF${}_{\mathrm{JAD}*}$ with frame-wise accuracy of 0.92, 0.89 F1-score and 0.06 Brier score (Table 2). RF${}_{\mathrm{JAD}*}$ uses 300 decision trees with a maximum depth of 80 without bootstrapping, 1 minimum sample per leaf and a minimum number of 2 samples to split a tree. The quality of a split is measured as a function of the information gain.

**Figure 5:**
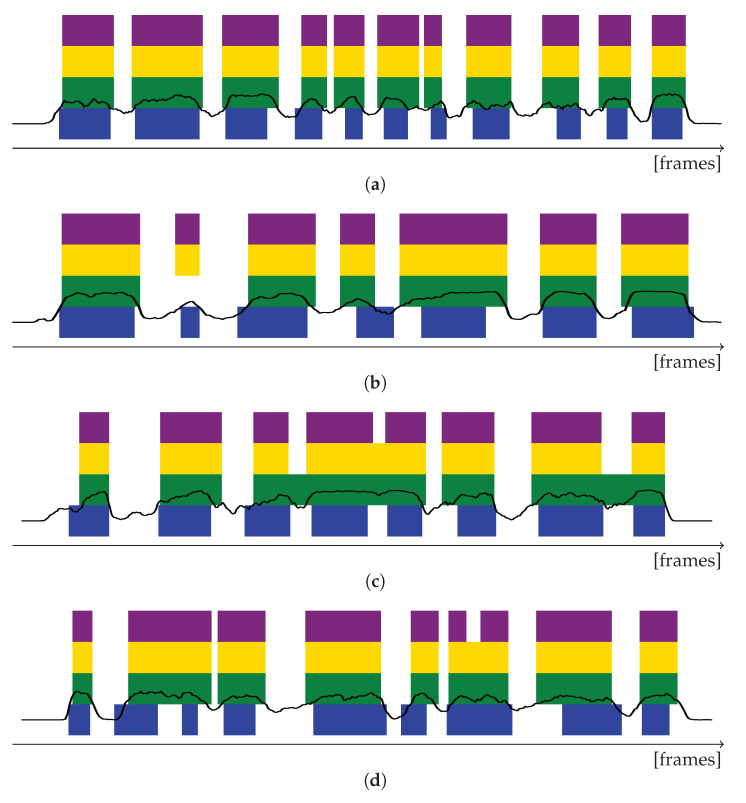
Sample segmentation results for 4 different sentences (**a**–**d**). Blue: Ground truth segments. Black: Confidence curves *c* of the automatic segmentation without smoothing. Green: segmentation $sp_{smg}$ obtained from strong Gaussian smoothing of $sp_{ini}$. Yellow: $sp_{med}$ obtained from retrieving smoothed out local peaks. Purple: split proposal $sp_{fin}$ as used for final evaluation in this manuscript.

Visual comparison to the manually annotated ground truth segments reveals that $sp_{ini}$ already provides many robust segmentation results as the one given in Figure 5a. Besides, the proposed post-processing step improved the validity of imperfect split proposals. First post-processing leading to $sp_{smg}$ can successfully recover significant parts that were smoothed out (Figure 5b), whereas second post-processing can additionally identify local minima above the binary threshold (Figure 5c) The impact of all remaining errors (e.g., an erroneous split caused by the lowest minimal peak being located above the binary threshold, see Figure 5d) should be analyzed after segment-wise classification in the context of full-sentence prediction errors.

### 5.2. Stage 2 Word Classification

The present task is a standard multi-class recognition problem that can be trained using the standard cross-entropy loss. Training of all classifiers was set to 2000 epochs with a dropout rate of 0.5 and a learning rate dynamically adapted via the Adam optimizer. As for the word segmentation, we evaluate the training processes as average of all 8 cross-validation folds and report the performance of the investigated classifiers with respect to accuracy, precision, recall and F1 score.

**Figure 6:**
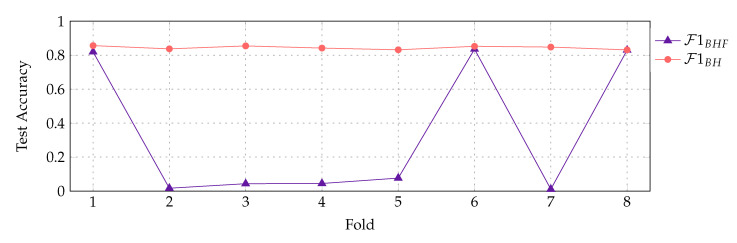
Classifier training under the BHF feature combination is much less stable than classifier training under the BH feature combination only (here: $F1_{BHF}$ and $F1_{BH}$ for L2).

As exemplary shown for L2 in Figure 6, a stable classification model cannot be learned under the BHF feature combination throughout the cross-validation folds. For BH on the other hand, a classifier with test accuracy >0.8 can be learned in all folds. This holds true for all three feature representations and both word class labels. Besides, in cases of successful classifier training, test accuracy of the BHF feature combination classifier slightly ranks below the BH feature combination classifier. This might suggest that fewer relevant information could be learned under the BHF feature combination. For this reason, all subsequent evaluation will be discussed utilizing the feature representations $F1_{BH}$, $F2_{BH}$ and $F3_{BH}$ only. The respective index terms will be omitted for simplicity in the following.

For all folds, training converges quickly under all three feature transformations. However, accuracy and loss reach their final value faster for F2 as compared to F1, and faster for F3 as compared to F2. Listing the average values of all evaluation metrics (Table 3), we see that F3 shows the best performance, and that F2 performs better than F1 under both L1 and L2. This observation suggests that an inter-chain based data representation is better suited to carry relevant information than a pure single reference point one: both F2 and F3 utilize angular representations of neighboring (and most often kinematic related) joints. Additional transformation of F3 into identically-sized three-dimensional images further seems to enhance structures or hidden information within a signing pattern that could not be retrieved otherwise.

**Figure 7:**
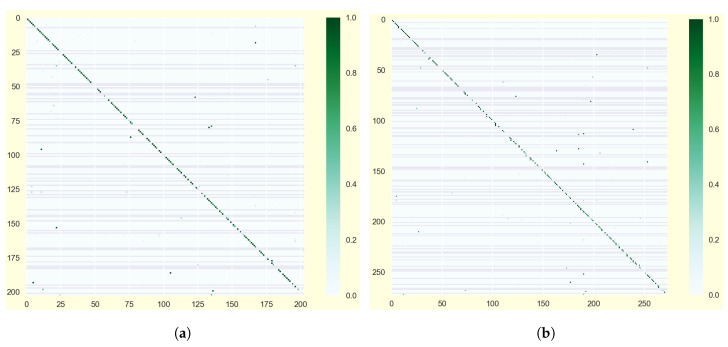
Exemplary confusion matrices for (**a**) CNN${}_{F3,L1}$ and (**b**) CNN${}_{F3,L2}$.

Next, we examine classifier performance differences with respect to the distinct representation of word classes. As expected, overall performance of the classifier trained under label set L2 is lower than for the one trained under the less specific label set L1. We can register a drop in the average accuracy of −5.75% and a drop of −6.67% in the average F1 score for F3. Similar performance decrease also exists for F1 (−5.75% accuracy and −6.33% F1) and F2 (−6.80% accuracy and −7.97% F1), whereas the relative decrease in performance of F3 and F2 is smaller than for F1. This confirms the previous assumption that the kinematic-induced angular representations contain more significant information for discrimination between different motion classes. Moreover, the general observed drop in accuracy under L2 conveys that the additional linguistic feature identification adds difficulty to the general recognition task. Grouping closely related words in consecutive order (as e.g., in the class sequence ‘tasty – tasty (CP2) – tasty (CP3)’), prediction patterns show a relatively high number of word confusions of closely related signs with and without NME. These become obvious in larger, indifferent clusters, as shown around the diagonal of the confusion matrices in Figure 7. Consequently, mostly morphological similar or even identical signs are affected by misclassification.

## 6. Final System Output

We combine the predictions of the CNN classifier into sequential output estimates. Based on the index of occurrence of every classified word segment within a respective test sentence we concatenate the labels to a final output. We then compare the resulting sentence predictions to their ground truth label and compute the WER of all test sentences. We compute the WER following its common definition as

(3) $$WER=\frac{s+d+i}{n}$$

with *s* being the number of necessary substitutions, *d* being the number of necessary deletions, *i* being the number of necessary insertions per word sequence, and *n* being the number of words in the reference sentence. We then determine the overall WER as average over all test data as given in the 8 cross validation fold splits.

Due to the staged system architecture, sequence prediction errors can be the cause of two different error sources. These are wrong temporal segmentation and wrong word segment classification. Comparison between the ground truth reference sentence and the outputs of the different classifier should provide a more detailed information on the main error source. In concrete, sentence parts that are poorly recognized throughout all CNN variations are very likely to be caused by segmentation errors. Sentences whose sub-parts are poorly recognized by single CNN variations are very likely to be caused by inferior classifier performance. To evaluate whether the classifiers trained under the word classes defined by L2 show sensitivity towards the underlying NMEs, we furthermore define two ground truth predictions GT${}_{L1}$ and GT${}_{RG}$ for every sequence. For GT${}_{L1}$, we simply determine the accuracy of all CNN${}_{F3,L1}$ output predictions with respect to the target sequences of L2. This means a prediction of the word ‘tasty’ is considered misclassified if the respective target label is ‘tasty (CP2)’ or ‘tasty (CP3)’. To determine GT${}_{RG}$, we randomly replace every possible NME item within the CNN${}_{F3,L1}$ predictions with any of the lexically related available choices. A prediction of the word ‘tasty’ is hence randomly labeled as either ‘tasty’, ‘tasty (CP2)’ or ‘tasty (CP3)’ and then compared to the target. Here, the idea is to determine the approximate baseline accuracy that could be obtained while guessing the content of any NME-loaded sign.

### 6.1. Results

For all CNN combinations, the average WER corresponds to the performance of the class-based word recognition. In particular, the WER of the NME-sensitive word class set L2 is higher than for the general set of word classes L1. This is observed throughout all types of data input F1, F2 and F3 (Table 4). F3 furthermore has the lowest, and hence best, WER. In total, we achieve a WER of 11.36% for L1, and a WER of 15.75% for L2. This compares to a WER of 28.97% for GT${}_{L1}$ (absolute improvement 13.22%) and 30.59% for GT${}_{RG}$ (absolute improvement 14.84%). Here, GT${}_{L1}$ achieves better results than GT${}_{RG}$ because the distribution of adjective inflection is slightly skewed towards the neutral word class. In conclusion, the proposed system and trained classifiers are clearly superior in identification and understanding of the non-manual information than non-specific classifier and random guessing. For a more detailed analysis, we will next have a closer look into the final sentence predictions of four sample sentences and their respective output predictions per data representation and label class set.

The first two sentence patterns A and B (Table 5) show successful prediction samples. They furthermore demonstrate the difference in WER as a consequence of the two sets of word classes, and their use as evaluation targets. For sentence pattern A, we can see that the reference contains two linguistic features, namely ‘pt1 (Gen.)’ and ‘tasty (CP2)’. Utilizing L1, the sentence is classified correctly under all network models. Under L2, F1 and F2 fail to identify one of the two linguistic structures correctly. F3 on the other hand identifies both non-verbal structures and achieves a perfect WER score. Sentence pattern B constitutes a question, and hence contains one non-verbal expression at the end of the sentence. We can see that F1 and F2 – in contrast to F3 – successfully recognize ‘pt3?’ as a question. However instead, they fail to classify ‘woman’ correctly. Consequently all feature variations are of same WER. Since we consider sentences without full speech content, it is difficult to further judge the impact of both errors. In both cases, WERs for the two ground truth sequences are higher than for the classifier trained under L2.

The third sentence pattern C (Table 6) gives an example for an imperfect output that is likely to be caused by erroneous segmentation. Specifically, the reference sentence contains the phrase ‘bell/tree/man’ which stands for ‘Mr. Suzuki’: the sign for ‘Suzuki’ is formed by the gestures for ‘bell’ (suzu) and ‘tree’ (ki). Since these two movements are very distinct, we consider them as separate signs in our temporal data annotation. However, none of the architecture variations classifies the consecutive word of ’bell’ as ‘tree’. This indicates that the two words might not have been split during segmentation. Instead, they might have been treated as a single word segment, which subsequently got classified as the longer and more distinct sub-part ‘bell’. Since the two signs constitute one word semantically, it appears likely that the signing was very fluent with fast transition, so that the segmentation failed to split the two words accordingly. Errors like the previous one might be easily post-processed before the final system output to reduce their impact. In other words, the final sequence prediction of ‘bell/man’ could also be treated as ‘Mr. Suzuki’, once ‘Mr. Bell’ is deemed an impossible combination.

Finally, sentence pattern D (Table 6) gives an example for a failed sentence prediction of high WER throughout all classifier variations. Two segmentation errors appear to be the main cause for the high WERs: an erroneous split of the sign ‘always’, as well as two missing splits of the three consecutive signs ‘go4’. As we can see, the word succeeding the correctly classified ‘always’ is mislabeled by all CNN variations, whereas the on average best classifier F3 classifies it as ‘always’ again. This is a strong indicator for the word erroneously being split in two parts. F2 and F3 on the other hand again correctly classify the phrase combination ‘camping/CP (P)’ succeeding the last of the three ‘go4’ within the reference sentence. We assume this repetitive pattern to be exceptionally difficult for the proposed system. First, ‘go4’ is a particular morphological modification of the basic sign ‘go’ utilized to express a group of people moving somewhere together. Second, to emphasize the larger number of people, it is signed repeatedly with alternating hands in a very fast and fluent manner. This makes it very difficult to separate between movements even in video-based visual annotation. For the implementation of a future system, it might hence be reasonable to treat multiple occurrences of single signs as one word, once they are quickly signed in a consecutive manner and convey a single semantic meaning. Such procedure should also not affect the understanding of the final sentence content.

## 7. Discussion

We proposed a staged classifier and evaluated its performance quality for the recognition of continuous sentence expressions with non-manual content in Japanese SL. The staged procedure is aligned with initial works of CSLR, but contradicts current evolution of speech and audio recognition that are all based on end-to-end approaches. The main motivation for our work was to progress system development sensitive to subtle, non-manual linguistic content specific for SLs. Our system achieves promising results in this context. The classifiers trained on L2 are able to correctly recognize NME-sensitive word labels with much higher average accuracy than insensitive prediction or random guessing. This particularly holds true for sentence predictions with stable and precise sentence segmentation, indicating that the conveyed subtle non-manual content can indeed be learned from the underlying joint movement data. This is particular interesting since the final input data constitutes of 2D joint information of the body and finger only. Inclusion of facial joint data made the classifier training unstable and did not improve results. Neural classifier training is commonly able to extract relevant information from the underlying data without manual feature engineering. However, poor data structure or a high number of irrelevant features might impede classifier training. As such, information on facial joint movement might be helpful to improve classifier accuracy under different data arrangements. For example, feature reduction methods like Principal Component Analysis could help to select only those facial features that would be relevant. In this work, we focused on the basic architecture and system design to explore CLSR with NME context. Respective work on how to better include facial information should be conducted in the future.

Although many NMEs are heavily reliant on changes in facial expressions, results suggest that the main characteristics of the linguistic feature information could already be learned from variations of the body movement only, such as differences in head trajectory. This observation should be very interesting and valuable in the future and help to reduce the overall number of necessary sign tracking features. Moreover, our results considerably outperform results reported for an end-to-end approach utilizing a reduced number of sentence collections of the same data set [31]. As such, the staged approach appears meaningful for small, sparse and insufficient data sets with high NME content, which are otherwise hard to learn.

For the moment, our system has only been trained and tested on Japanese SL and a selection of 203 lexical items, respectively 273 distinct sign items including NMEs. As such, it is difficult to evaluate its ability to generalize onto other SLs or an extended corpus of sign words. However, since the proposed word segmentation is independent of the content of the underlying signed expression, we are confident that the proposed system can easily be extend to a larger number of signed words, once a respective data set is made available.

To put our work into context with existing works, it would next be necessary to compare our method with different data sets and state-of-the-art architectures. However, a balanced comparison can be hard to make. For one reason, the main goal of our work was not to improve a general system WER, but to explore whether a system that can better handle and understand non-manual linguistic features left rather unexplored to date. Second, our system targets Japanese SL, whose intrinsic linguistic information—due to cultural differences—is often less distinct than related features in Western SLs. As a consequence, it appears difficult to directly relate the system to any previous learning architecture, or·to examine it under baseline data like the SIGNUM or PHOENIX data set. In comparison to the neural network architecture by Ye et al. [26], whose purpose and underlying NME-heavy data is most similar to ours, our sentence recognition demonstrates superior performance. Whereas Ye et al. reach 69.2% recognition accuracy for 27 ASL words, we are able to classify 273 items with 86% accuracy (Table 3). This might indicate the usability of our approach for subsequent development of communication assist systems within Japan.

To the best of our knowledge, this is the first time the recognition of non-verbal expressions within continuous sign sequences has been investigated in detail with respect to different feature and network variations. We discovered that in many cases it is possible to correctly recognize additional linguistic features such as questions, adjective inflection and contextual references. For all network variations, WERs of the NME-sensitive set of word classes is approximately 5% higher than for the word class set that only differentiates signs on the base of their lexical manual morphology. Results show that kinematic-induced data transformations (F2, F3) are helpful to achieve a higher classification accuracy. As previously reported, transformation into JADMs of identical shape (F3) brings a further gain in accuracy. Most recently, further data transformation strategies for activity or sign recognition were reported [5,41]. These might result in even better recognition results once applied within the proposed pipeline. Oppositely, training of the word classification network converges quickly. This suggests the existence of a maximal accuracy level that is determined by errors in the sentence segmentation, and that might not be surpassed under the proposed two-staged strategy. To improve WER rates, it might therefore be helpful to modify the underlying sentence annotations used for supervised learning of the segmentation step. Ideally this would be achieved in collaboration with native speakers of SL. In such way, a more robust ground truth label could be obtained and the impact of errors (such as the ones in sample pattern D of Section 6.1) could be reduced. Lastly, it should be considered that many of the extended word classes were trained on a very small number of data only. We are confident to be able to distinguish respective non-verbal information with even higher accuracy once more training data is available.

## 8. Conclusions

We implemented a novel staged system for Continuous Sign Language Recognition, whose ability to understand complex linguistic content was evaluated with a set of signed video sequences in Japanese Sign Language. The system constitutes of two main processing steps, a first automatic temporal segmentation with a binary Random Forest classifier, and a segment-wise word classification of a Convolutional Neural Network. Both steps are learned in a supervised fashion and rely on basic ground truth data obtained from manual video annotation. The fundamental system input data are post-processed two-dimensional joint angular trajectories of body, finger and facial joints extracted from a signer’s video data using the open source library OpenPose. The performance of the system was evaluated under different types of data transformations and two different sets of word class labels. We used a set of general, lexical-item word classes that only distinguishes non-manual sign morphology, and a more complex and specific set of word classes that includes various linguistic non-manual features. The best data transformations achieve high accuracy for both the segmentation step (frame-wise accuracy 0.92) and the segment classification step (accuracy 0.91). This results in an overall average sentence WER of 11.36% for the lexical-item only class labels and 15.71% for the NME-sensitive class labels. As compared to predictions insensitive to non-manual features or made under random guessing, we achieve an improvement between 13.22% to 14.84%, suggesting that NMEs can well be learned within the separate word segments.

In a next step, we will investigate our system with additional data, and under specific settings where communication assist technologies are particularly useful, such as work meetings and assemblies. By this, we hope to make the learned classifiers even more robust, and to be able to further explore structures necessary for the reliable detection of non-verbal information within signed expressions. Lastly, we aim to adapt our two-staged architecture to different Sign Languages, as well as data sets of other movement types from the activity recognition field. As such, this work could then contribute to the future development of continuous sign communication assist tools with high usability and reliability under linguistically complex signed expressions. Moreover, successful modification could also assist in the development of collaborative machines that better understand and estimate human behavior.

## Figures and Tables

**Table 1:** Different Butterworth filter settings are used to smooth the joint position data obtained using OpenPose.

Joint	Cutoff Freq.	Filter Order
hips, knees, head (top)	1 Hz	1
shoulders, head (center)	1 Hz	2
elbows	6 Hz	2
fingers	6 Hz	2
face	6 Hz	2
wrists	8 Hz	3

**Table 2:** Binary classification performance of different RF models averaged over an 8-fold cross-validation. The Brier score was only determined for the fine-tuned JAD* model.

	Precision	Recall	F1	Accuracy	Brier
RF${}_{\mathrm{LS}}$	0.83	0.89	0.86	0.89	nA
RF${}_{\mathrm{LS}-\mathrm{IS}}$	0.84	0.89	0.87	0.90	nA
RF${}_{\mathrm{JAD}}$	**0.86**	**0.91**	**0.88**	**0.91**	nA
RF${}_{\mathrm{JAD}*}$	***0.86***	***0.92***	***0.89***	***0.92***	***0.06***

**Table 3:** Segment classification performance for combinations of feature and label sets averaged over an 8-fold cross-validation.

	Precision	Recall	F1	Accuracy
CNN${}_{F1,L1}$	0.85	0.86	0.85	0.86
CNN${}_{F2,L1}$	0.86	0.88	0.86	0.88
CNN${}_{F3,L1}$	**0.90**	**0.91**	**0.90**	**0.91**
CNN${}_{F1,L2}$	0.79	0.81	0.79	0.81
CNN${}_{F2,L2}$	0.81	0.83	0.81	0.83
CNN${}_{F3,L2}$	**0.85**	**0.86**	**0.85**	**0.86**

**Table 4:** Average WER of all test sentence output estimates obtained through 8-fold cross validation for the differently trained CNNs with respect to their respective target word class set.

	F1	F2	F3
L1	17.83%	15.71%	11.36%
L2	22.76%	20.13%	15.75%

**Table 5:** Successful sample predictions as obtained from the different classifiers in comparison to their reference (REF) and the two ground truths GT${}_{L1}$ and GT${}_{RG}$. NMEs in REF are highlighted by underlining.

	Content	WER
REF A	**pt1(Gen.)**/mother/pt3/cafe/CP (P)/**tasty(CP2)**/banana/cake/eat/end/pt3	
GT${}_{L1}$	pt1/mother/pt3/cafe/CP (P)/tasty/banana/cake/eat/end/pt3	18.18%
GT${}_{RG}$	pt1(Gen.)/mother/pt3/cafe/CP (P)/tasty/banana/cake/eat/end/pt3	9.09%
CNN${}_{F1,L1}$	pt1/mother/pt3/cafe/CP (P)/tasty/banana/cake/eat/end/pt3	0.00%
CNN${}_{F2,L1}$	pt1/mother/pt3/cafe/CP (P)/tasty/banana/cake/eat/end/pt3	0.00%
CNN${}_{F3,L1}$	pt1/mother/pt3/cafe/CP (P)/tasty/banana/cake/eat/end/pt3	0.00%
CNN${}_{F1,L2}$	pt1(Gen.)/mother/pt3/cafe/CP (P)/tasty/banana/cake/eat/end/pt3	9.09%
CNN${}_{F2,L2}$	pt1/mother/pt3/cafe/CP (P)/tasty(CP2)/banana/cake/eat/end/pt3	9.09%
CNN${}_{F3,L2}$	pt1(Gen.)/mother/pt3/cafe/CP (P)/tasty(CP2)/banana/cake/eat/end/pt3	0.00%
REF B	Sato/man/teach/woman/flower/present/purpose/CP (S)/CP (BL)/go/past/**pt3?**	
GT${}_{L1}$	Sato/man/teach/woman/flower/present/purpose/CP (S)/CP (BL)/go/past/pt3	8.33%
GT${}_{RG}$	Sato/man/teach/woman/flower/present/purpose/CP (S)/CP (BL)/go/past/pt3	8.33%
CNN${}_{F1,L1}$	Sato/man/teach/woman/flower/present/purpose/CP (S)/CP (BL)/pt3/past/pt3	8.33%
CNN${}_{F2,L1}$	Sato/man/teach/woman/flower/present/purpose/CP (S)/CP (BL)/go/past/pt3	0.00%
CNN${}_{F3,L1}$	Sato/man/teach/woman/flower/present/purpose/CP (S)/CP (BL)/go/past/pt3	0.00%
CNN${}_{F1,L2}$	Sato/man/teach/man/flower/present/purpose/CP (S)/CP (BL)/go/past/pt3?	8.33%
CNN${}_{F2,L2}$	Sato/man/teach/pt2/flower/present/purpose/CP (S)/CP (BL)/go/past/pt3?	8.33%
CNN${}_{F3,L2}$	Sato/man/teach/woman/flower/present/purpose/CP (S)/CP (B)/go/past/pt3	8.33%

**Table 6:** Sample outputs suffering from segmentation errors as obtained from the different classifiers in comparison to their reference (REF) and the two ground truths GT${}_{L1}$ and GT${}_{RG}$. NMEs in REF are highlighted by underlining.

	Content	WER
REF C	pt3/movie/CP (BL)/pt3/pt1/bell/tree/**man(Gen.)**/wife/CP (2ppl)/interesting/movie/watch/past	
GT${}_{L1}$	pt3/movie/CP (BL)/pt3/pt1/bell/ man/wife/despite/interesting/movie/watch/past	21.43%
GT${}_{RG}$	pt3/movie/CP (BL)/pt3/pt1/bell/ /man(Gen.)/wife/despite/interesting(CP3)/movie/watch/past	21.43%
CNN${}_{F1,L1}$	pt3/movie/CP (BL)/pt3/pt1/bell/ man/wife/CP (2ppl)/interesting/movie/watch/past	7.14%
CNN${}_{F2,L1}$	pt3/movie/CP (BL)/pt3/pt1/bell/ man/wife/CP (2ppl)/interesting/movie/watch/past	7.14%
CNN${}_{F3,L1}$	pt3/movie/CP (BL)/pt3/pt1/bell/ man/wife/despite/interesting/movie/watch/past	14.29%
CNN${}_{F1,L2}$	pt3/movie/CP (BL)/pt3/pt1/bell/ man/wife/CP (2ppl)/interesting/movie/watch/past	14.29%
CNN${}_{F2,L2}$	pt3/movie/CP (BL)/pt3/pt1/bell/ man/wife/CP (2ppl)/interesting/movie/watch/past	14.29%
CNN${}_{F3,L2}$	pt3/movie/CP (BL)/pt3/pt1/bell/ man/wife/go/interesting/movie/watch/past	21.43%
REF D	pt2/always/amuse/go4/go4/go4/camping/CP (P)/pt3/**good(CP3)**/**same?**	
GT${}_{L1}$	pt2/always/always/amuse/man/camping/CP (P)/pt3/good/same?	54.55%
GT${}_{RG}$	pt2/always/always/amuse/man/camping/CP (P)/pt3/good(CP2)/same?	45.45%
CNN${}_{F1,L1}$	pt2/end/break/amuse/man/camping/man/pt2/good/same	63.64%
CNN${}_{F2,L1}$	pt2/always/man/amuse/man/camping/CP (P)/pt2/good/same	45.45%
CNN${}_{F3,L1}$	pt2/always/always/amuse/man/camping/CP (P)/pt3/good/same	36.36%
CNN${}_{F1,L2}$	pt2/end/lover/amuse/man/camping/pt3/bell/good/same?	72.73%
CNN${}_{F2,L2}$	pt2/always/man/amuse/party/camping/CP (P)/pt2/good/same?	54.55%
CNN${}_{F3,L2}$	pt2/always/always/amuse/man/camping/CP (P)/pt3/good/same?	45.45%

## Abbreviations

The following abbreviations are used in this manuscript:

SL	Sign Language
JSL	Japanese Sign Language
CSLR	Continuous Sign Language Recognition
NME	Non-Manual Expression
CP	Classifier Predicates
WER	Word Error Rate
RF	Random Forest
CNN	Convolutional Neural Network
B	Body (joint features)
H	Hand (joint features)
F	Face (joint features)
